# Age at Menarche Among Mizo Women in Aizawl, Mizoram: A Secular Trend Across Five Birth-Decade Cohorts (1960-2007)

**DOI:** 10.7759/cureus.106523

**Published:** 2026-04-06

**Authors:** Lalhruaia Ralte, Lalnuntluanga Sailo, Lalmalsawma Hnamte, Benjamin Lalrinpuia, C Biakhlupuii, Vanlalduhsaki Ralte

**Affiliations:** 1 Department of Physiology, Zoram Medical College and Hospital, Aizawl, IND; 2 Department of Respiratory Medicine, Zoram Medical College and Hospital, Aizawl, IND; 3 Department of Community Medicine, Zoram Medical College and Hospital, Aizawl, IND

**Keywords:** age of menarche, birth cohort study, early menarche, female reproductive health, mizo, mizoram, north east india, puberty onset, secular trend, urbanization trends

## Abstract

Background: Age at menarche (AAM) is a key indicator of female pubertal development. A secular decline in AAM has been observed globally, but data from indigenous populations of Northeast India remain limited. This study documents the secular trend in AAM among Mizo ethnic women over five decades and examines its association with pre-menarcheal residential setting.

Methods: A cross-sectional retrospective study was conducted among 2,178 Mizo women currently residing in Aizawl and born between 1960 and 2007, classified into five birth-decade cohorts. AAM was obtained through structured bilingual questionnaires. Pre-menarcheal residential setting (rural or urban) was based on self-reported childhood residence. Linear regression was the primary analysis; one-way ANOVA and nonparametric tests served as confirmatory analyses. Multivariable regression and bootstrap resampling were used to test sensitivity to confounding and unequal cohort sizes.

Results: The overall mean AAM was 13.50±1.49 years. A significant declining trend was observed across cohorts (F(4,2173)=54.587, p<0.001, η²=0.091): mean AAM decreased from 14.67±1.19 years (1960-1969) to 13.09±1.45 years (2000-2007), a decline of approximately 0.40 years per decade. When restricted to the three most recent cohorts (1980-2007), where recall intervals were shorter, the decline remained significant (0.30 years per decade). Women with rural pre-menarcheal residence had later AAM than those with urban residence (13.76 vs. 13.34 years; Cohen’s d=0.280), though the effect was small. In multivariable regression, the year-of-birth effect remained significant after covariate adjustment (B=-0.034, p<0.001). The prevalence of early menarche (≤12 years) increased from 4.0% to 35.2% across cohorts (p<0.001).

Conclusions: A significant secular decline in AAM has occurred among Mizo women in Aizawl over five decades, consistent across analytical approaches and residential strata. The concurrent rise in early menarche prevalence carries public health relevance given Mizoram’s high noncommunicable disease burden. Although generalizability to rural Mizoram is limited, these findings add to the limited evidence on menarcheal timing among indigenous ethnic groups in Northeast India and underscore the need for prospective studies linking pubertal timing to later-life disease outcomes.

## Introduction

Menarche, the onset of the first menstrual period, is a defining event in female puberty and a widely used marker of reproductive maturation. It reflects the interplay of genetic predisposition, nutritional status, body composition, and socioeconomic conditions [1-3]. Secular trends in age at menarche (AAM) have been studied extensively across populations globally, with the most consistent finding being a progressive decline in AAM over the twentieth century, attributed largely to improvements in nutrition and living standards [4,5]. This trend has continued in many low- and middle-income countries [6].

In Western populations, secular declines of approximately three to four months per decade were reported in the early to mid-twentieth century, with the trend largely plateauing by the 1960s to 1980s [4]. In contrast, developing nations, including India, have demonstrated continued and more recent downward trends in AAM [7-9]. National-level Indian data have documented mean AAM values ranging from 12.5 to 14.5 years, with urban populations consistently exhibiting earlier menarche compared with their rural counterparts [7,9]. However, these data derive predominantly from Indo-Aryan and Dravidian populations of mainland India, and indigenous ethnic groups of Northeast India, a region that is genetically and culturally distinct, remain substantially underrepresented in the literature.

The Mizo are an indigenous Tibeto-Burman people of East Asian origin, belonging to the Kuki-Chin linguistic branch, who inhabit the state of Mizoram in Northeast India. Genomic studies confirm that the Mizo carry predominantly East Asian genetic lineages distinct from mainland Indian populations [10,11], and reproductive health parameters derived from Indo-Aryan and Dravidian groups may not be applicable. Since Mizoram attained statehood in 1987, the population has undergone rapid socioeconomic transformation, including urbanization, improved access to education and healthcare, and dietary transitions from traditional to market-imported foods [12], conditions that are well-established drivers of secular change in AAM [3,5]. Despite this, no dedicated study of menarcheal timing has been published for the Mizo population.

Documenting secular trends in AAM among the Mizo is important for several reasons. First, it provides baseline reproductive health data for an underrepresented indigenous ethnic group. Second, because AAM varies across ancestral populations, national-level estimates may not generalize to ethnically distinct subpopulations, and population-specific data are therefore needed. Third, there is clinical urgency: each year of earlier menarche has been associated with increased risk of cardiovascular disease, type 2 diabetes, and breast cancer [13-15], and Mizoram already has the highest age-adjusted cancer incidence rate in India [16]. Understanding whether AAM is declining in this population, and how rapidly, is therefore relevant to emerging non-communicable disease patterns in the region.

The present study therefore aimed to: (i) document the secular trend in AAM across five birth-decade cohorts among Mizo ethnic women born between 1960 and 2007; (ii) examine the association between pre-menarcheal residential setting (rural vs. urban) and AAM, including how the residential distribution of women has changed over cohorts; and (iii) assess the prevalence of early menarche (≤12 years) across cohorts and residential strata.

## Materials and methods

Study design and setting

A cross-sectional retrospective study was conducted among Mizo ethnic women residing in the Aizawl Municipal Corporation (AMC) area, Mizoram, Northeast India. The term "cohort" is used throughout to denote birth-decade groups within this cross-sectional sample, classified retrospectively by year of birth, and does not imply prospective longitudinal follow-up. The AMC comprises 19 wards and 87 local councils. Institutional ethical approval was obtained from the Institutional Ethics Committee, Zoram Medical College and Hospital (Approval No. F.20016/1/18-ZMC/IEC/136, dated December 10, 2024) before commencement of data collection. Data were collected between March and October 2025 after obtaining informed written consent from all participants.

Study population and sampling

The target population comprised Mizo ethnic women currently living in Aizawl. Inclusion criteria were: (i) females aged 18 years and above; (ii) current residents of the AMC area. Exclusion criteria were: (a) either parent not of Mizo ethnicity; (b) had not attained menarche; (c) unable to recall AAM; (d) internally inconsistent or implausible responses; (e) born before 1960; (f) AAM reported as after 18 years; (g) self-reported history of chronic illness, prolonged medication use, or disability during childhood that could affect pubertal timing. Women born after 2007 were ineligible under inclusion criterion (i), as they would have been younger than 18 years at the time of data collection. Women born in 2007 were included if they had attained 18 years of age at the time of questionnaire administration. The traditional upper age limit for diagnosing primary amenorrhea is 16 years in the presence of secondary sexual characteristics [17]; however, because the present study includes women born as early as the 1960s, when population-level AAM was substantially higher than in contemporary cohorts, the more conservative cutoff of 18 years was used for exclusion criterion (f) to avoid excluding late-normal maturers from earlier birth cohorts.

The minimum sample size per cohort was calculated using the formula for comparing two independent means: $n=\frac{(Z_{\alpha/2}+Z_{\beta})^2 \times 2\sigma^2}{\Delta^2}$, where Zα/2=1.96 (α=0.05, two-tailed), Zβ=0.84 (80% power), σ=1.5 years (a conservative estimate based on the largest cohort-specific SD of 1.40 years reported by Meher and Sahoo [18]), and Δ=0.44 years (the overall inter-cohort difference in mean menarcheal age reported in the same study [18]). This yielded a target minimum of 182 participants per cohort. Since the study required representation across five birth-decade cohorts, the total sample was scaled to ensure adequate representation of the smallest cohort by population share (women born 1960-1969, aged 56-65 years at the time of data collection), who constitute approximately 8.83% of the adult female population based on 2021 population projections for Northeast India [19]. The required total sample was therefore estimated as 182÷0.0883=2,061. An inflation factor of 20% was applied to account for anticipated non-response, incomplete data, and ineligibility (2,061×1.20=2,473, rounded to 2,475). A design effect correction was not applied, as no prior Mizo data were available to estimate an intraclass correlation coefficient (ICC) for AAM. In the absence of published ICC estimates for AAM in any Mizo or comparable Tibeto-Burman population, applying an assumed design effect would have been arbitrary; a post hoc sensitivity analysis across a plausible ICC range is reported in the Limitations.

A two-stage cluster sampling design was employed. Using the lottery method, nine of the 19 wards within the AMC were randomly selected, and one local council was randomly chosen from each selected ward, yielding nine primary sampling units. Within each selected local council, trained research assistants visited consecutive households, briefed eligible women on the purpose and procedures of the study, and distributed questionnaires to all eligible women present. All eligible women present in each household at the time of the visit were invited to participate. Non-response characteristics were not formally assessed beyond the overall response rate (93.5%). A total of 275 questionnaires were allocated to each of the nine selected local councils, totaling 2,475.

Participants were classified into five birth-decade cohorts based on year of birth: Cohort 1 (1960-1969), Cohort 2 (1970-1979), Cohort 3 (1980-1989), Cohort 4 (1990-1999), and Cohort 5 (2000-2007). Cohort 5 spans eight years rather than 10 owing to the minimum age eligibility criterion of 18 years at the time of data collection; however, its sample size (n=764) is the largest of all cohorts, ensuring adequate statistical power.

Data collection

Data were collected using a structured, self-administered bilingual questionnaire (English and Mizo, Appendix 1). The questionnaire was developed by the authors for this study and is provided in the Appendices. The primary outcome variable was self-reported AAM in completed years, consistent with the methodology of comparable Indian population-based surveys [7,18]. Participants were asked to recall the age at which they experienced their first menstrual period and were explicitly instructed not to guess if they could not recall their AAM. The questionnaire was pilot tested among approximately 30 women from non-sampled local councils within the AMC to assess clarity and comprehension of bilingual items; minor wording adjustments were made based on feedback. Formal validation was not undertaken, as the questionnaire records objective biographical facts rather than subjective perceptions or attitudes. No additional structured recall-anchoring techniques were employed; the implications of recall bias are addressed in the Limitations.

Pre-menarcheal residential setting was classified as rural or urban based on the participant’s self-reported predominant place of residence during childhood prior to menarche. Because the same locality may have transitioned from rural to urban across the study period, each reported place of residence was manually verified against its Census classification during the participant’s pre-menarcheal years, using the Census closest to the relevant period (the most recent being the 2011 Census of India). Duration of residence at each setting was not recorded. The highest parental education level was defined as the higher of the mother’s or father’s education and was recorded on a 7-point ordinal scale (1=illiterate, 2=primary school, 3=middle school, 4=high school, 5=higher secondary, 6=graduate, 7=postgraduate or above).

Statistical analysis

Descriptive statistics (mean, SD, median, and range) were computed for AAM overall, by birth-decade cohort, and by residential setting. Distributional normality was assessed using skewness and kurtosis indices; values within ±2 were considered indicative of approximate normality, consistent with recommendations for large samples where formal tests (e.g., Shapiro-Wilk) are overly sensitive.

The prespecified primary analysis was simple linear regression with AAM as the outcome and year of birth as the predictor, to quantify the secular trend. One-way ANOVA with Tukey HSD post hoc pairwise comparisons, the Kruskal-Wallis test, and the Jonckheere-Terpstra test served as prespecified confirmatory analyses of the cohort-level differences. An independent-samples t-test was used to compare AAM between rural and urban groups. Effect sizes were reported as eta-squared (η²) for ANOVA, and as Cohen’s d for pairwise and between-group comparisons. No additional correction for multiple comparisons was applied across confirmatory analyses, as these test the same hypothesis from complementary angles; the Tukey HSD (Honestly Significant Difference) procedure controls the family-wise error rate for pairwise comparisons. Pearson’s correlation coefficient was computed to assess the linear association between year of birth and AAM.

To examine the role of residential setting, a two-way ANOVA was used to test the interaction between birth-decade cohort and residential setting on AAM, and stratified linear regression analyses were performed within each residential stratum. Levene’s test was used to verify the homogeneity of variances. A hierarchical multivariable linear regression was performed to determine whether the secular trend persisted after adjusting for residential setting (dummy-coded: rural=0, urban=1) and parental education. Parental education was entered as a continuous covariate; a sensitivity analysis treating it as a categorical variable (six dummy variables) was also performed to test the linearity assumption.

The prevalence of early menarche (defined as AAM ≤12 years [13,14]) was calculated by cohort and residential setting, and the chi-square test for linear trend (linear-by-linear association) was used to assess changes in prevalence across cohorts. Recall accuracy was assessed through cohort-stratified frequency analysis of reported AAM to check for digit preference or heaping. Participants with missing data on specific variables were excluded from the relevant analyses rather than the entire study.

To assess whether unequal cohort sizes distorted the secular trend estimate, inverse-cohort-size weighted regression and bootstrap resampling (10,000 iterations, cohort-stratified) were performed as post hoc sensitivity analyses. Equal-size resampling (all cohorts resampled to n=100; 5,000 iterations) was also performed. All analyses were performed using IBM SPSS Statistics version 27 (IBM Corp., Armonk, NY), with the exception of bootstrap resampling and weighted regression, which were performed in Python 3 (NumPy and SciPy libraries) (Python Software Foundation, Wilmington, DE). A two-tailed significance level of p<0.05 was used throughout. This study is reported in accordance with the STROBE guidelines for cross-sectional studies.

## Results

Sample characteristics

A total of 2,475 questionnaires were distributed, of which 2,314 were returned (93.5% response rate; 161 unreturned). Of these, 136 were excluded: non-Mizo ethnicity (n=44), unable to recall AAM (n=6), implausible responses (e.g., reported AAM preceding the year of birth; n=3), born before 1960 (n=24), AAM reported as after 18 years (n=8), and self-reported chronic illness, prolonged medication use, or disability during childhood (n=51). No participants were excluded for not having attained menarche or for being born after 2007. The final analytical sample comprised 2,178 eligible women. No missing data were present for the primary outcome variable (AAM) or year of birth.

The distribution across birth-decade cohorts was as follows: Cohort 1 (1960-1969), n=100 (4.6%); Cohort 2 (1970-1979), n=242 (11.1%); Cohort 3 (1980-1989), n=373 (17.1%); Cohort 4 (1990-1999), n=699 (32.1%); and Cohort 5 (2000-2007), n=764 (35.1%).

**Figure 1:**
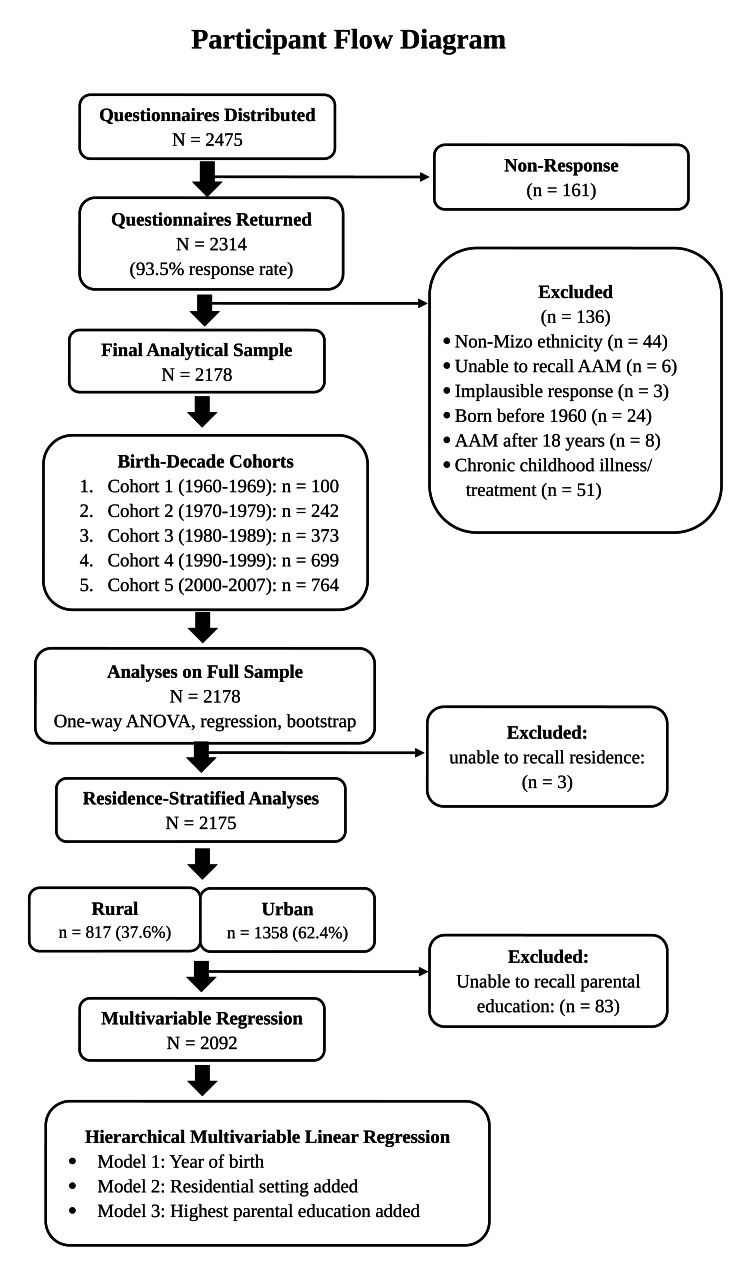
Participant flow diagram AAM, age at menarche

Three participants (0.1%) could not recall their pre-menarcheal residence and were excluded from residence-stratified analyses (valid N=2,175). Of the remaining 2,175, 817 (37.6%) were from rural areas and 1,358 (62.4%) from urban areas. Additionally, 83 participants (3.8%) were unable to recall parental education level; together with the three participants missing residence data, a total of 86 were excluded from the multivariable regression (valid N=2,092). Among the 2,092 participants with valid parental education data, the mean highest parental education level increased across cohorts, from 2.53 in Cohort 1 to 4.16 in Cohort 5. The participant flow is summarized in Figure 1.

Secular trend in age at menarche

**Figure 2:**
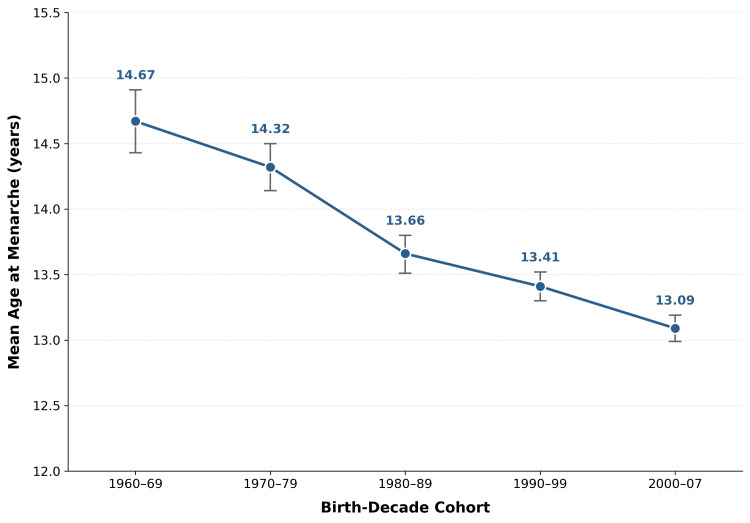
Mean AAM by birth-decade cohort (N=2,178) Error bars represent 95% CI. AAM, age at menarche

**Figure 3:**
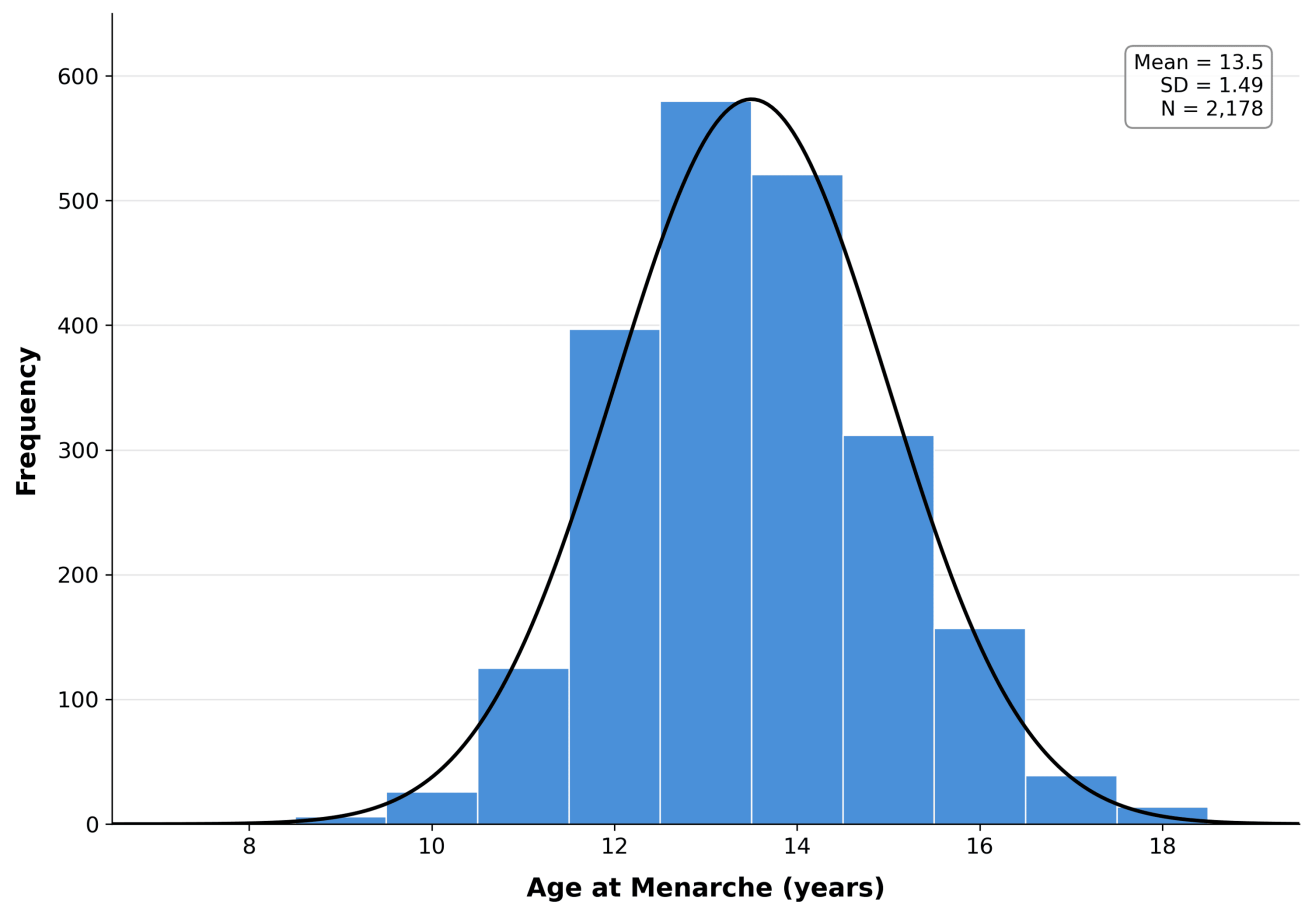
Distribution of AAM The superimposed curve represents the normal distribution. AAM, age at menarche

Mean AAM declined progressively across all five cohorts (Table 1; Figure 2). The distribution was approximately normal (skewness=0.183, kurtosis=0.091; Figure 3), with an overall mean of 13.50±1.49 years (median: 13.0; range: 8-18). Mean AAM decreased from 14.67±1.19 years in Cohort 1 to 13.09±1.45 years in Cohort 5, a total reduction of 1.58 years.

**Table 1:** AAM (years) by birth-decade cohort (N=2,178) AAM, age at menarche

Cohort	n	Mean	SD	Median	Min	Max	95% CI
1960-1969	100	14.67	1.19	15	12	17	14.43-14.91
1970-1979	242	14.32	1.42	14	10	18	14.14-14.50
1980-1989	373	13.66	1.4	14	8	18	13.51-13.80
1990-1999	699	13.41	1.45	13	10	18	13.30-13.52
2000-2007	764	13.09	1.45	13	9	18	12.99-13.19

Levene’s test confirmed homogeneity of variances (F=0.800, p=0.525). One-way ANOVA revealed a statistically significant difference in AAM across the five cohorts (F(4,2173)=54.587, p<0.001, η²=0.091). The nonparametric Kruskal-Wallis test confirmed this finding (H(4)=208.435, p<0.001). The Jonckheere-Terpstra test demonstrated a statistically significant ordered declining trend across cohorts (Z=-13.379, p<0.001). Cohen’s d between the earliest and latest cohorts (1960-1969 vs. 2000-2007) was 1.11, indicating a large effect size.

Tukey HSD post hoc pairwise comparisons are presented in Table 2. Significant differences were observed between all non-adjacent cohort pairs (all p<0.001). Among adjacent pairs, the 1970s vs. 1980s (p<0.001) and 1990s vs. 2000s (p<0.001) comparisons were significant, while the 1960s vs. 1970s (p=0.242) and 1980s vs. 1990s (p=0.058) were not.

**Table 2:** Tukey HSD post hoc pairwise comparisons of AAM between birth-decade cohorts *The mean difference is significant at the 0.05 level. ^†^CI lower bound is slightly negative (-0.01), consistent with the non-significant p-value. AAM, age at menarche; SE, standard error; HSD, Honestly Significant Difference

Comparison	Mean Diff (years)	SE	p-value	95% CI
1960s vs. 1970s	0.35	0.17	0.242	-0.12 to 0.81
1960s vs. 1980s	1.01*	0.161	<0.001	0.57 to 1.45
1960s vs. 1990s	1.26*	0.152	<0.001	0.84 to 1.67
1960s vs. 2000s	1.58*	0.152	<0.001	1.17 to 2.00
1970s vs. 1980s	0.67*	0.118	<0.001	0.34 to 0.99
1970s vs. 1990s	0.91*	0.106	<0.001	0.62 to 1.20
1970s vs. 2000s	1.23*	0.105	<0.001	0.95 to 1.52
1980s vs. 1990s	0.24	0.091	0.058	-0.01 to 0.49^†^
1980s vs. 2000s	0.57*	0.09	<0.001	0.32 to 0.81
1990s vs. 2000s	0.32*	0.075	<0.001	0.12 to 0.53

Trend quantification

Simple linear regression with year of birth as the predictor yielded a slope of -0.040 years per calendar year (95% CI: -0.046 to -0.035; p<0.001; R²=0.087; adjusted R²=0.087), corresponding to a decline of approximately 0.40 years per decade. The Pearson correlation coefficient between year of birth and AAM was r=-0.296 (p<0.001). Stratified bootstrap resampling (10,000 iterations) supported the stability of this estimate, yielding a mean slope of -0.040 years per year (bootstrap 95% CI: -0.046 to -0.035), with 100% of iterations producing a negative slope. Inverse-cohort-size weighted regression yielded a slope of -0.043 years per year (0.43 years per decade). Equal-size resampling of all cohorts to n=100 (5,000 iterations) produced a mean slope of -0.043 years per year (95% CI: -0.051 to -0.034). As a further sensitivity analysis, restricting the sample to the three most recent cohorts (1980-2007; n=1,836) yielded a slope of -0.030 years per year (0.30 years per decade, p<0.001).

Association of residential setting with age at menarche

Women with rural pre-menarcheal residence had significantly later AAM than those with urban residence (Table 3). An independent-samples t-test (equal variances not assumed, Levene's F=4.090, p=0.043) demonstrated a statistically significant difference (t(1811.882)=6.428, p<0.001; Cohen's d=0.280), indicating a small effect size.

**Table 3:** AAM (years) by pre-menarcheal residential setting (N=2,175) Three participants who were unable to recall pre-menarcheal residence were excluded. Cohen's d=0.280 (95% CI: 0.193, 0.367). AAM: age at menarche

Residence	n (%)	Mean	SD	Median	t-test	Mean diff (95% CI)
Rural	817 (37.6)	13.76	1.42	14	t=6.428, p<0.001	0.414 (0.288, 0.541)
Urban	1,358 (62.4)	13.34	1.52	13		

The proportion of women residing in urban areas before menarche increased across successive cohorts: 40.0% in Cohort 1 (1960-1969), 51.5% in Cohort 2, 60.3% in Cohort 3, 65.6% in Cohort 4, and 67.0% in Cohort 5 (2000-2007).

Interaction between cohort and residence

**Figure 4:**
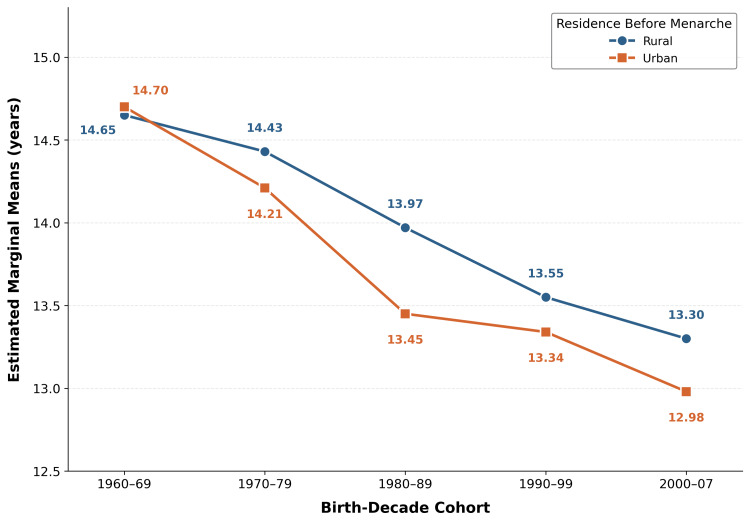
Estimated marginal means of AAM by birth-decade cohort and residential setting Both strata show parallel downward trends, consistent with the non-significant cohort×residence interaction (p=0.348). AAM, age at menarche

The secular decline was parallel across residential strata, with no significant cohort x residence interaction. Levene’s test confirmed homogeneity of variances (F(9,2165)=1.444, p=0.164). Two-way ANOVA revealed that both main effects remained significant after mutual adjustment: birth-decade cohort (F(4,2165)=48.567, p<0.001, partial η²=0.082) and residential setting (F(1,2165)=9.092, p=0.003, partial η²=0.004). The interaction between cohort and residence was not statistically significant (F(4,2165)=1.115, p=0.348, partial η²=0.002), indicating that the secular decline in AAM did not differ significantly between women with rural and urban pre-menarcheal residence (model R²=0.101, adjusted R²=0.098). The estimated marginal means by cohort and residence are illustrated in Figure 4.

Stratified linear regression analyses demonstrated that the secular decline in AAM was statistically significant within both residential strata (Table 4).

**Table 4:** Stratified linear regression of year of birth on AAM by residential setting The overall regression includes all 2,178 participants regardless of residence status; stratified rows exclude three participants unable to recall pre-menarcheal residence (valid N=2,175). AAM, age at menarche; B, unstandardized regression coefficient; β, standardized regression coefficient

Residence	n	B	β	95% CI for B	R²	p	Decline/decade
Rural	817	-0.037	-0.317	-0.045 to -0.030	0.1	<0.001	0.37
Urban	1,358	-0.039	-0.262	-0.047 to -0.031	0.069	<0.001	0.39
Overall	2,178	-0.040	-0.296	-0.046 to -0.035	0.087	<0.001	0.40

Multivariable regression analysis

The year-of-birth effect persisted after sequential adjustment for residential setting and parental education (Table 5). The year-of-birth coefficient attenuated from -0.041 (Model 1, unadjusted) to -0.034 (Model 3, fully adjusted) but remained statistically significant (p<0.001). The slight difference between the Model 1 slope (B=-0.041) and the primary regression slope (B=-0.040, N=2,178) reflects the smaller complete-case sample (N=2,092). In the full model, each one-unit increase on the seven-point parental education scale was associated with 0.136 years earlier menarche, and the model explained 10.6% of the total variance in AAM. A sensitivity analysis treating parental education as a categorical variable yielded a virtually identical year-of-birth coefficient (B=-0.035 vs. -0.034) and comparable model fit (R²=0.108 vs. 0.106), confirming that the linearity assumption did not bias the secular trend estimate.

**Table 5:** Hierarchical multivariable linear regression of AAM on year of birth, residential setting, and parental education (N=2,092) Residential setting dummy-coded (rural=0, urban=1). Parental education entered as continuous (seven-point ordinal scale). *p<0.05; **p<0.01; ***p<0.001 AAM, age at menarche; B, unstandardized regression coefficient; R², coefficient of determination; F change, F-statistic for the significance of R² change

Predictor	Model 1	Model 2	Model 3
	B (95% CI)	B (95% CI)	B (95% CI)
Year of birth	-0.041	-0.040	-0.034
	(-0.047 to -0.035)	(-0.046 to -0.034)	(-0.041 to -0.028)
Residential setting (urban)	-	-0.303***	-0.178**
		(-0.431 to -0.175)	(-0.313 to -0.043)
Parental education	-	-	-0.136***
			(-0.186 to -0.086)
R²	0.085	0.094	0.106
Adjusted R²	0.084	0.093	0.105
R² change	-	0.009	0.012
F change	-	21.502***	28.484***
N	2,092	2,092	2,092

Prevalence of early menarche

**Figure 5:**
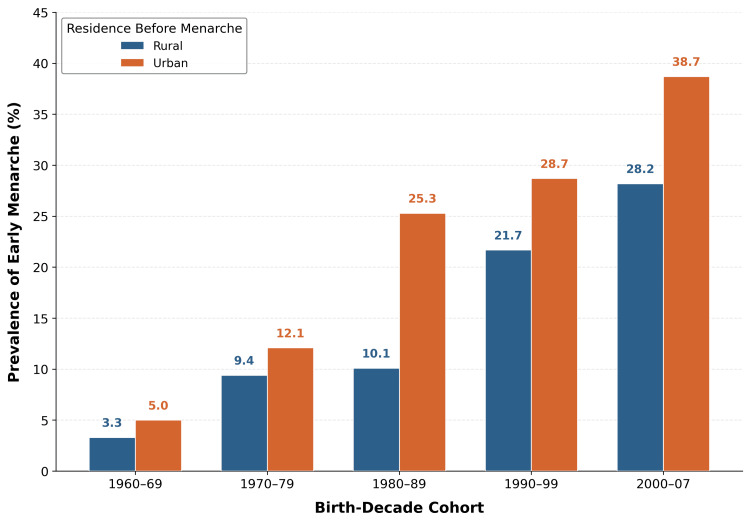
Prevalence of early menarche (≤12 years) by birth-decade cohort and residential setting

The prevalence of early menarche (≤12 years) increased significantly across successive birth-decade cohorts in both residential strata (Table 6; Figure 5), from 4.0% (4/100) in Cohort 1 to 35.2% (269/764) in Cohort 5 (linear-by-linear association=97.195, p<0.001): rural (3.3% to 28.2%, linear-by-linear=37.376, p<0.001) and urban (5.0% to 38.7%, linear-by-linear=50.200, p<0.001). The overall prevalence of early menarche in the study population was 25.5% (555/2,178). As a sensitivity analysis using a stricter threshold (≤11 years), the prevalence increased from 0.0% in Cohort 1 to 11.6% in Cohort 5. The full frequency distribution of reported AAM by birth-decade cohort is presented in Table 7.

**Table 6:** Prevalence of early menarche (≤12 years) by birth-decade cohort and residential setting Cohort totals differ slightly from Table 1 because residence-stratified prevalence excludes three participants unable to recall pre-menarcheal residence (valid N=2,175). Linear-by-linear association: overall=97.195, p<0.001; rural=37.376, p<0.001; urban=50.200, p<0.001. ^†^Small cell size; interpret with caution

Cohort	Total (n)	Early menarche (n)	%	Rural, n/N (%)	Urban, n/N (%)
1960-1969	100	4	4	2/60 (3.3)	2/40 (5.0)^†^
1970-1979	241	26	10.8	11/117 (9.4)	15/124 (12.1)
1980-1989	373	72	19.3	15/148 (10.1)	57/225 (25.3)
1990-1999	697	183	26.3	52/240 (21.7)	131/457 (28.7)
2000-2007	764	269	35.2	71/252 (28.2)	198/512 (38.7)

**Table 7:** Frequency distribution of reported AAM (years) by birth-decade cohort (N=2,178) Values shown are n (% within cohort). AAM, age at menarche

AAM	1960-1969	1970-1979	1980-1989	1990-1999	2000-2007	Total
8	0 (0.0)	0 (0.0)	1 (0.3)	0 (0.0)	0 (0.0)	1 (0.0)
9	0 (0.0)	0 (0.0)	0 (0.0)	0 (0.0)	6 (0.8)	6 (0.3)
10	0 (0.0)	1 (0.4)	2 (0.5)	8 (1.1)	15 (2.0)	26 (1.2)
11	0 (0.0)	5 (2.1)	12 (3.2)	40 (5.7)	68 (8.9)	125 (5.7)
12	4 (4.0)	20 (8.3)	57 (15.3)	136 (19.5)	180 (23.6)	397 (18.2)
13	12 (12.0)	38 (15.7)	106 (28.4)	212 (30.3)	212 (27.7)	580 (26.6)
14	28 (28.0)	60 (24.8)	103 (27.6)	164 (23.5)	166 (21.7)	521 (23.9)
15	29 (29.0)	78 (32.2)	50 (13.4)	82 (11.7)	73 (9.6)	312 (14.3)
16	23 (23.0)	27 (11.2)	35 (9.4)	36 (5.2)	36 (4.7)	157 (7.2)
17	4 (4.0)	9 (3.7)	6 (1.6)	13 (1.9)	7 (0.9)	39 (1.8)
18	0 (0.0)	4 (1.7)	1 (0.3)	8 (1.1)	1 (0.1)	14 (0.6)
Total	100	242	373	699	764	2,178

## Discussion

The present study documents a significant and sustained secular decline in AAM among Mizo ethnic women over five decades, with mean AAM decreasing from 14.67 years in women born in the 1960s to 13.09 years in those born between 2000 and 2007, a reduction of 1.58 years. This trend was consistent across parametric, nonparametric, and bootstrap approaches, and persisted after adjustment for pre-menarcheal residential setting and parental education. Additionally, women with rural pre-menarcheal residence exhibited modestly later AAM than their urban counterparts, though the secular decline was parallel across both strata. The prevalence of early menarche (≤12 years) rose from 4.0% to 35.2% over the study period, and even using a stricter threshold of ≤11 years, the prevalence increased from 0.0% to 11.6%.

The rate of decline of approximately 0.40 years per decade is substantially faster than the approximately one month per decade reported by Pathak et al. [7] from national-level Indian data, although direct comparison is complicated by differences in methodology and population coverage. Bajpai et al. [8], in a prospective study among North Indian girls that included a review of 21st-century Indian data, reported a secular decline of approximately 0.41 years per decade, closely comparable to the present findings. For reference, historically reported secular declines of three to four months per decade in Western European populations during the early to mid-twentieth century have largely plateaued since the 1960s [4]. The continued decline through the most recent cohort (2000-2007) suggests that the secular trend has not yet plateaued in this population. The assumption of linearity is supported by convergent findings from model-free analyses (Jonckheere-Terpstra and Kruskal-Wallis tests); the restricted-cohort analysis (1980-2007) yielded a somewhat attenuated slope (0.30 vs. 0.40 years per decade), which may suggest modest deceleration in more recent cohorts or may reflect the removal of older cohorts with longer recall intervals.

Comparing the present findings with national and international cohort data helps place the magnitude of the secular decline in a broader context. The Indian Human Development Survey placed the national mean AAM at 13.83 years for women born in 1955-1964 [7]. In Western Europe, mean AAM had already declined to approximately 13-13.5 years among women born in the 1950s, by which point the secular trend was beginning to plateau in several countries [4]. Against this backdrop, the mean AAM of 14.67 years in the earliest Mizo cohort (1960-1969) was notably higher than both the national Indian average and the Western European range for the same period, suggesting that the Mizo population entered the secular transition from a comparatively high baseline. This is consistent with the relatively late onset of socioeconomic modernization in Mizoram, where the post-statehood acceleration of improvements in nutrition, healthcare access, and educational attainment [12,20] may have accelerated what would otherwise have been a more gradual transition. By the most recent cohort (2000-2007), the Mizo mean AAM of 13.09 years has converged toward the national trajectory, falling below the all-cohort pooled NFHS mean of 13.49 years (covering birth cohorts from approximately 1942 to 2006) [18].

Within the context of Northeast India, the region has consistently demonstrated the lowest mean AAM in the country. Meher and Sahoo [18], pooling NFHS data across three survey rounds, reported a mean AAM of 12.62±1.27 years for the Northeastern region, the lowest of all six Indian regions. Among studies of comparable birth cohorts in Northeast India, reported mean AAM values range from 13.22 years in Khasi girls [21] and 13.72 years in Chakma girls [22] to 12.5-13.1 years in Meitei women of Manipur [23], with earlier Assamese studies reporting 11.83-11.94 years [24,25], placing the Mizo 1990-1999 cohort mean of 13.41 years within the middle range of regional ethnic variation. The mean AAM of 13.09 years in the most recent Mizo cohort (2000-2007) is approaching, though still above, the regional NFHS estimate of 12.62 years [18], suggesting convergence toward the broader Northeastern pattern. These findings provide population-specific secular trend data for the Mizo that were previously unavailable, complementing the limited ethnic-specific evidence from this region.

Given the Mizo’s Tibeto-Burman linguistic and genetic heritage, the only available large-scale comparator from a related population group is the study by Liu et al. [26], who examined 1.275 million women born between 1965 and 2001 across 26 ethnic groups in Yunnan Province, China, a region that includes several Tibeto-Burman speaking groups alongside Han and other ethnicities. They reported a pooled mean AAM of 13.7 years and a secular decline of 0.36 years per decade, broadly comparable to the Mizo findings. Direct comparison is limited, however, because the Yunnan estimate reflects all 26 ethnic groups, including the Han majority, which likely dominates the pooled figure, rather than Tibeto-Burman populations specifically. Liu et al. did not report ethnic subgroup-specific estimates, precluding a more precise comparison.

Women with rural pre-menarcheal residence had statistically later AAM (13.76 years) than those with urban residence (13.34 years), consistent with the rural-urban gradient documented across India [7,9]. However, the effect size was small (Cohen’s d=0.280), suggesting that the statistical significance may partly reflect the large sample size. This is consistent with a broader national pattern: an analysis of 25 years of Indian DHS data found that the rural-urban regression coefficient for AAM declined from 0.213 in 1992 to 0.030 in 2019 [9], indicating that the rural-urban gap in menarcheal timing has narrowed substantially. The secular decline was observed at nearly identical rates within both residential strata (Table 4), and the non-significant cohort x residence interaction (p=0.348) indicates that the trend is not driven by the growing proportion of women with urban pre-menarcheal residence in later cohorts (40.0% in the 1960s to 67.0% in the 2000s), although the small cell sizes in early cohorts (e.g., Cohort 1 urban n=40) may limit power to detect a modest interaction.

The socioeconomic changes that have characterized post-statehood Mizoram [12,20] may have contributed to the observed decline. Childhood body mass index and adiposity are well-established proximal determinants of menarcheal timing [2,3]; however, pre-menarcheal anthropometric data were not collected in the present study (see Limitations), and improved childhood nutritional conditions therefore remain a possible but untested hypothesis. Parental education, included as a socioeconomic proxy, independently predicted earlier AAM in the multivariable model, and its inclusion attenuated the year-of-birth coefficient only modestly (from -0.041 to -0.034 years per year). This suggests that intergenerational improvements in education account for a portion of the secular change but leave a substantial residual trend attributable to unmeasured factors, which may include dietary transition toward higher protein and fat intake, exposure to endocrine-disrupting chemicals, and psychosocial stress, none of which were assessed in the present study. The low overall R² (0.106 in the full model) is expected for a multifactorial trait at the population level; the primary interest of this analysis is the magnitude and significance of the secular trend slope rather than the proportion of variance explained.

The rising prevalence of early menarche (≤12 completed years) from approximately 4% in the 1960s cohort to approximately 35% in the 2000s cohort indicates that the secular decline is not limited to a shift in mean values but represents a redistribution of the population toward younger menarcheal ages, with public health implications. This pattern held even at a stricter threshold: the prevalence of menarche at ≤11 years increased from 0.0% to 11.6% over the same period. This upward trend mirrors findings from other populations: in the United States, the prevalence of early menarche rose from 8.6% to 15.5% across birth cohorts spanning 1950 to 2005 [27]; nationally in India, 17.2% of women report early menarche, with the proportion reaching 48.6% in the Northeastern region, the highest of all Indian regions [18]; and a recent study in Karnataka found that 33% of adolescent girls experienced menarche before age 12 [28]. Early menarche has been associated with increased risk of breast cancer, cardiovascular disease, type 2 diabetes, and psychosocial difficulties in adolescence [13-15]. Mizoram’s emerging non-communicable disease burden adds urgency to this finding: the state has the highest age-adjusted cancer incidence rate in India, with Aizawl district reporting an age-adjusted rate of 214.1 per 100,000 in women during 2012-2016 [16], and breast cancer incidence in Aizawl is the highest of all population-based cancer registries in Northeast India [29]. While the present study cannot establish a causal link between declining AAM and these health outcomes, the concurrent trends are consistent with the epidemiological literature linking early menarche to long-term metabolic and reproductive health risks [13-15] and point to the need for public health surveillance that tracks pubertal timing alongside chronic disease indicators.

Limitations

Several limitations should be acknowledged. First, AAM was obtained by retrospective self-report, which is vulnerable to recall bias, particularly among older women recalling events from several decades earlier. The direction of any recall-related bias would be toward overestimation of AAM in older cohorts, and therefore overestimation of the secular decline. The magnitude of this potential inflation is bounded by the restricted-cohort analysis (1980-2007), which yielded a decline of 0.30 years per decade (p<0.001) compared with 0.40 for the full sample, suggesting that recall-related inflation accounts for at most approximately 25% of the observed trend. Cohort-stratified frequency analysis showed no evidence of problematic digit preference or heaping, with no single age accounting for more than 32.2% of any cohort (Table 7), and previous validation studies have demonstrated acceptable reliability of recalled AAM in adult women [30,31]. Additionally, AAM was recorded in completed years, which may systematically underestimate true AAM by approximately 0.5 years on average, thereby inflating the prevalence of early menarche; however, this convention affects all cohorts equally, does not bias the secular trend estimate, and is consistent with the methodology of comparable Indian studies [7,18]. Survivorship bias is a related concern, as women born in the 1960s must have survived to ages 56-65 years to be interviewed. The direction of this bias is uncertain: if women with earlier menarche had higher mortality, their absence from the oldest cohort would inflate the cohort mean, again leading to overestimation of the decline. However, the direct evidence linking menarcheal timing to all-cause mortality below age 70 remains limited, and this concern diminishes progressively for younger cohorts.

Second, the cross-sectional retrospective design permits documentation of cohort differences but precludes causal inference regarding the specific drivers of the observed decline.

Third, childhood anthropometric data were not collected, as pre-menarcheal body composition cannot be measured retrospectively in adult women aged 18-65 years, and the role of childhood nutritional status in driving the decline could therefore not be examined. Parental education was used as a socioeconomic proxy, but this measure captures educational capital rather than material conditions, and the assumption of linearity across the seven-point scale, while supported by sensitivity analysis, remains an approximation.

Fourth, the pre-menarcheal residential setting was recorded as a self-reported binary variable (rural or urban), manually verified against Census classification, but with duration of residence unrecorded; this variable should be interpreted as a broad indicator of childhood developmental context rather than a precise measure of exposure.

Fifth, all participants were current residents of the AMC area; women who remained in rural Mizoram throughout their lives are not represented, and generalizability to the broader rural Mizo population should not be assumed. The direction of this selection bias is toward potential underestimation of the rural-urban difference in AAM, as women who migrated from rural Mizoram to Aizawl are classified as current urban residents but may have had rural childhood exposures. Although wards and local councils were selected by probability methods, participant-level sampling within each local council was systematic (consecutive household visits) rather than strictly random; however, the high response rate (93.5%) and coverage across nine geographically distributed clusters reduce the likelihood of substantial selection bias. Cohort 1 (1960-1969) comprised only 100 participants, below the calculated minimum of 182 per cohort; the overall secular trend, derived from continuous year-of-birth regression across all 2,178 participants, does not depend on the adequacy of any single cohort, but pairwise comparisons involving this cohort should be interpreted with caution.

Sixth, cluster identifiers were not retained in the analytical dataset, precluding computation of cluster-robust standard errors. A post hoc design effect sensitivity analysis assuming ICC values from 0.005 to 0.05 confirmed that the primary secular trend finding remained statistically significant across this range. The rural-urban difference, already small in effect size (Cohen's d=0.280), would require formal cluster adjustment to estimate with greater precision, and future studies should retain cluster identifiers to permit this.

## Conclusions

AAM among Mizo women in Aizawl has declined significantly over five decades, from 14.67 years in the 1960s cohort to 13.09 years in those born between 2000 and 2007. This secular decline was consistent across analytical approaches, persisted after covariate adjustment, and was observed in both rural and urban residential strata. Concurrently, the prevalence of early menarche rose from approximately 4% to 35%, indicating a population-wide redistribution toward younger menarcheal ages. These findings carry public health relevance given Mizoram’s high noncommunicable disease burden. Adolescent health programs in Mizoram should consider incorporating pubertal timing surveillance alongside existing nutritional and reproductive health monitoring. The present data may also serve as population-specific reference values for clinical assessment of menarcheal timing in Mizo girls. Future prospective studies incorporating childhood anthropometry and dietary data across both urban and rural Mizoram are needed to clarify the mechanisms driving this trend and to determine whether earlier menarche in this population is translating into measurable changes in later-life disease risk.

## Appendices

Appendix 1

Questionnaire on the age at menarche among Mizo women in Aizawl, Mizoram: a secular trend across five birth-decade cohorts (1960-2007)

Questionnaire (Original is in English and Mizo format)

1. Have you already turned 18 years old? (Yes/No)

2. Have you experienced menstrual bleeding? (Yes/Not yet)

3. Are both your parents Mizo? (Yes/No)

4. During your childhood, did you suffer from any prolonged illness, prolonged medication, chemotherapy, or hormone therapy? (Yes/No/Do not recall)

5. Year of birth (write exact year, e.g., 1984, 2005): __________

6. What is your blood group? (A+/A−/B+/B−/AB+/AB−/O+/O−/Do not know)

7. How old were you when you first had menstrual bleeding? __________

8. How heavy was your first menstrual bleed? (Light/Heavy/Do not recall)

9. How painful was it? (Not painful/Mildly painful/Very painful/Do not recall)

10. How many days did it bleed? (1-3 days/4-7 days/More than 7 days/Do not recall)

11. How long did it take to become regular? (Immediately/Within 1 year/2 years/3 years/More than 4 years/Do not recall)

12. Before your first menstrual bleed, where were you living? (Write name of village/town/city): __________

13. Before your first menstrual bleed, how physically active were you? (Not active/Moderately active/Very active/Do not recall)

14. Among your parents, who had higher education? (Mother/Father/Equal)

15. What was the highest education? (Illiterate/Primary School/Middle School/High School/Higher Secondary/Graduate/Postgraduate or above)
